# Vascularized scapula and latissimus dorsi flap for CAD/CAM assisted reconstruction of mandibular defects including the mandibular condyle: technical report and clinical results

**DOI:** 10.1186/s12893-019-0535-3

**Published:** 2019-06-26

**Authors:** Pit Jacob Voss, David Steybe, Marc Anton Fuessinger, Wiebke Semper-Hogg, Marc Metzger, Rainer Schmelzeisen, Philipp Poxleitner

**Affiliations:** 1 Department of Oral and Craniomaxillofacial Surgery, Center for Dental Medicine, University Medical Center Freiburg, Hugstetter Straße 55, 79106 Freiburg, Germany; 2 Berta-Ottenstein-Programme for Clinician Scientists, Faculty of Medicine, University of Freiburg, Freiburg, Germany

**Keywords:** Disarticulation resection, Reconstruction, Scapula flap, CAD/CAM

## Abstract

**Background:**

Reconstruction of mandibular continuity and function after tumor resection is challenging, particularly in cases including the mandibular condyle. Various approaches for reconstruction after disarticulation resection have been reported. However, the scapula flap has received little attention as a treatment option in these cases.

**Patients and methods:**

Three cases of computer aided design and computer aided manufacturing (CAD/CAM) assisted reconstruction after disarticulation resection using a vascularized scapula and latissimus dorsi flap are reported. All cases required reconstruction of the mandibular ramus and condyle in combination with the reconstruction of large and complex soft tissue defects.

**Results:**

The surgical procedure was deemed successful in all cases. The scapula flap could be placed as preoperatively planned and patients regained their preoperative occlusion pattern and satisfying mouth opening-ranges. The large soft tissue defects could reliably be reconstructed using a latissimus dorsi flap.

**Conclusions:**

The scapula and latissimus dorsi flap can be considered a suitable option for the reconstruction of mandibular disarticulation resection defects in combination with large soft tissue defects.

## Background

Continuity resection of the mandibular bone is indicated in a number of orofacial neoplasms. Dependent on the extent of mandibular involvement, resection of the ascending mandibular portion, including the mandibular condyle, can be necessary. This form of resection, during which condylar articulation is sacrificed, is referred to as disarticulation resection [1]. Reconstruction of the mandibular continuity is challenging, particularly in cases of disarticulation resection [2, 3]. Full mouth opening requires a complex movement with a combination of initial rotation followed by a translational movement of the mandibular condyle, making adequate reconstruction of this structure particularly important [4]. Failure of adequate reconstruction of the mandibular condyle, a major component of the temporomandibular joint (TMJ), can result in severe facial deformity, difficulty with chewing and subsequent severe reduction of the patient’s quality of life [3, 5].

A variety of techniques including alloplastic materials, autologous tissue or a combination of these both is available for the reconstruction of the mandibular ramus and condyle. The use of patient specific condylar titanium-prostheses has been reported by several authors [1, 2, 6–8]. However, this approach has yielded mixed results with potential complications being hardware failure/degradation, foreign body reactions, implant exposure or erosion of the cranial base [1]. Free vascular fibular flaps for mandibular reconstruction were introduced by Hidalgo in 1989 [9]. Since then, they have become a popular option for mandibular reconstruction and are considered the current gold standard by many authors [3, 10]. This flap currently represents the favored option for autologous reconstruction in cases of disarticulation resection as well [11].

The scapula tip with the latissimus dorsi muscle as a single free flap was first described by Coleman and Sultan in 1991 [12]. This flap possesses a number of advantages: The shape and surface contour of the scapular tip can be seen as the ideal option to restore the shape of the mandibular condyle [13]. Moreover, it provides a long vascular pedicle and flexible soft tissue paddle being ideal for the three-dimensional reconstruction of complex and extensive resection defects [14].

In the past, controlling the correct position of a free flap mandibular reconstruction was very difficult, as it relied solely on the surgeon’s experience and intraoperative visual examination. The progress of computer aided design (CAD) and computer aided manufacturing (CAM) has created the basis for superior outcomes in reconstructing complex mandibular defects [15, 16]. Applying CAD/CAM, it is now possible to perform exact preoperative planning with construction of cutting and drilling guides as well as patient-specific osteosynthesis plates.

In this study we investigated the feasibility of CAD/CAM planned reconstruction of disarticulation mandibular resection defects using a scapula and latissimus dorsi free flap. We were aiming at evaluating the accuracy of bone reconstruction as well as functional and esthetic outcomes to provide a basis for this technique to be investigated in a larger patient cohort.

## Patients and methods

Three patients with pathologies requiring resection of the mandibular ramus, condyle and significant amounts of soft tissue were included in the investigation. Pathologies were of neoplastic origin in patient 1 (recurrent liposarcoma of the left paroid gland) and patient 2 (basosquamous carcinoma of the right cheek) and radiation therapy related (osteoradionecrosis; ORN) in patient 3.

In all patients CAD/CAM planned reconstruction with a vascularized scapula flap was performed at the Department of Oral and Craniomaxillofacial Surgery, University Medical Center Freiburg, Germany. A scapula and latissimus dorsi flap was chosen due to the significant soft tissue involvement in all patients.

**Fig. 1:**
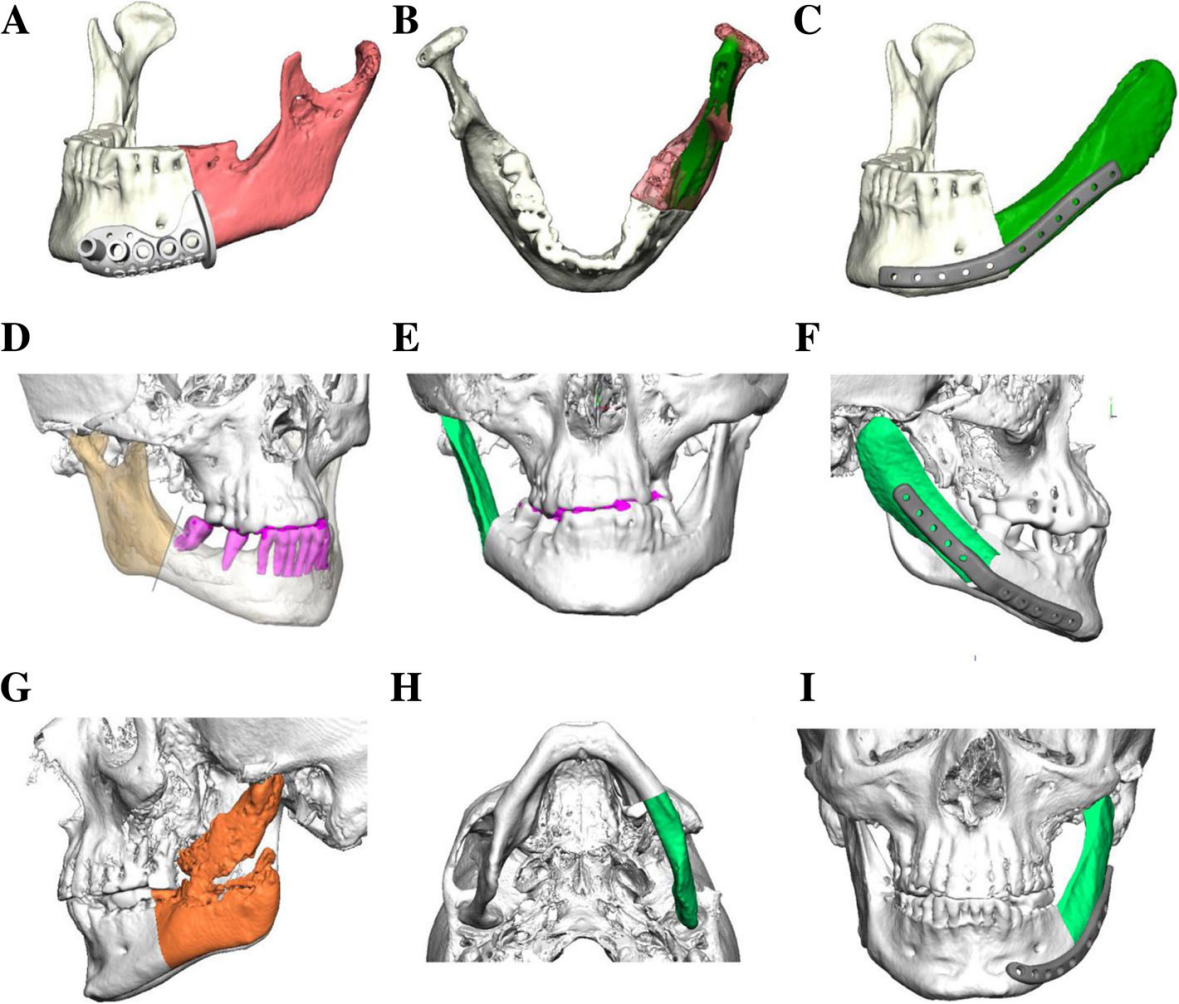
Visualization of CAD/CAM planning of patient 1 (**a-c**), patient 2 (**d-f**) and patient 3 (**g-i**). (**a, d, g**) depict the bony parts to be resected (colored parts); (**b, e, h**) depict the planned shape and localization of the scapula bone flap (colored in green); (**c, f, i**) visualize the shape and localization of the patient specific osteosynthesis plate

High resolution CT scans of the craniofacial region and the donor site (scapula) were acquired of each patient. Virtual three-dimensional reconstructions of the mandible and the scapula produced from this dataset represent the basis for the virtual planning of the surgical procedure (Fig. 1). This preoperative planning procedure is performed in a web-based conference in collaboration of the maxillofacial surgeon and a technician of the virtual planning vendor (KLS Martin Group, Tuttlingen, Germany). During this planning conference, key parameters like resection margins, placement of cutting and drilling guides and shape of the patient specific osteosynthesis plate are addressed. The finalized virtual plan is used for the production of cutting and drilling guides for the mandible and the scapula as well as a patient specific osteosynthesis plate (Fig. 1).

**Fig. 2:**
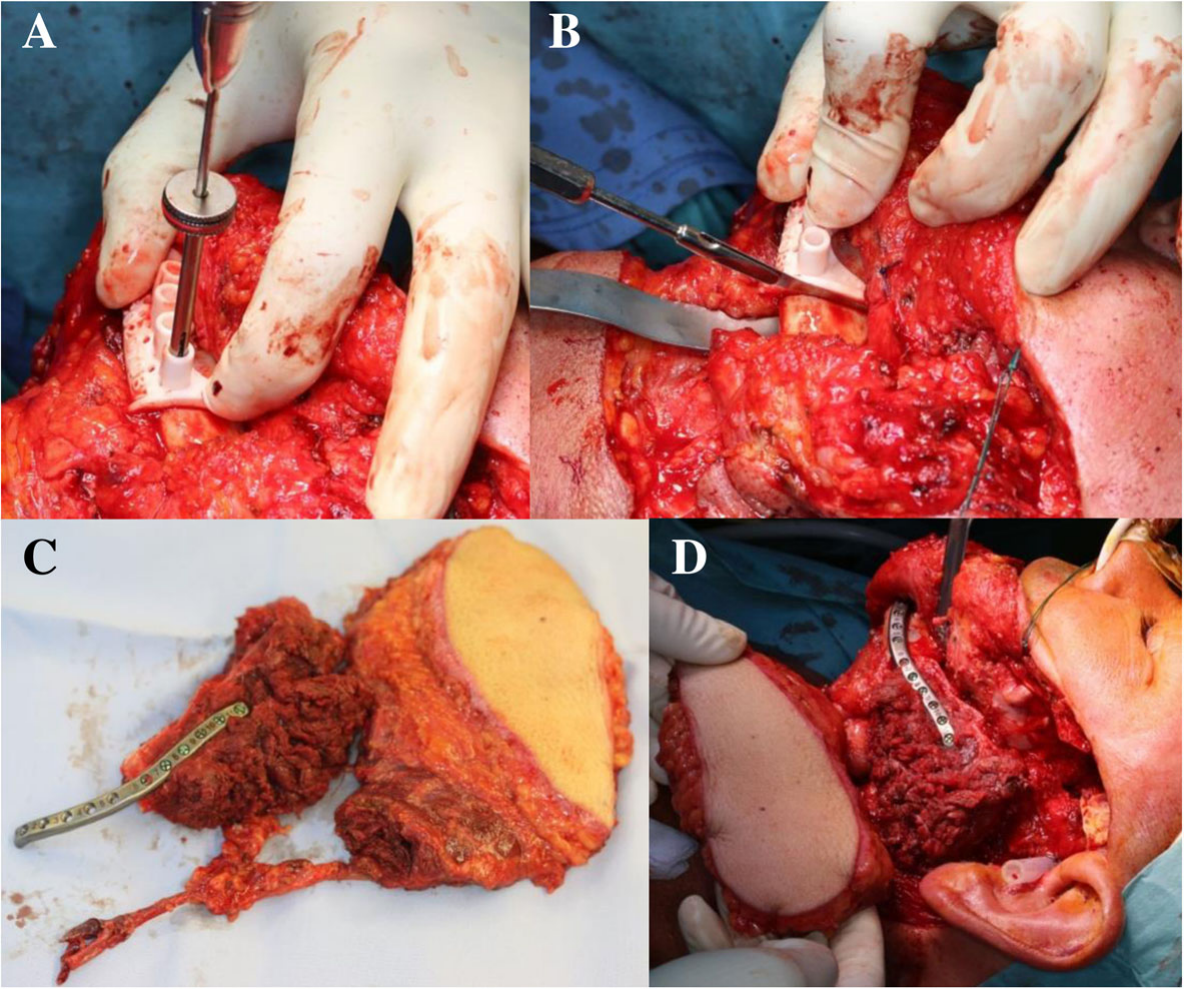
Surgical procedure with guided placement of drill holes (**a**) and guided mandibular resection (**b**). The resection guides are placed onto the parts of the mandible not to be resected. The patient-specific osteosynthesis plate is fixed to the scapula flap (**c**) with drill holes placed according to the drilling guide (not depicted) and can thus serve as a guidance for the placement of the flap (**d**)

Surgery is performed under general anesthesia with the patient being in mandibulo-maxillary fixation. Exposure of the jaw is achieved through a transcervical or submandibular approach; further approaches are added if necessary to fully access the site of surgery. Drill holes are placed using the guiding holes in the cutting guides (Fig. 2a). Subsequent resection of the mandible, including resection of the condyle from the glenoid fossa, is performed according to the preoperative planning using the CAD/CAM fabricated cutting guides (Fig. 2b).

**Fig. 3:**
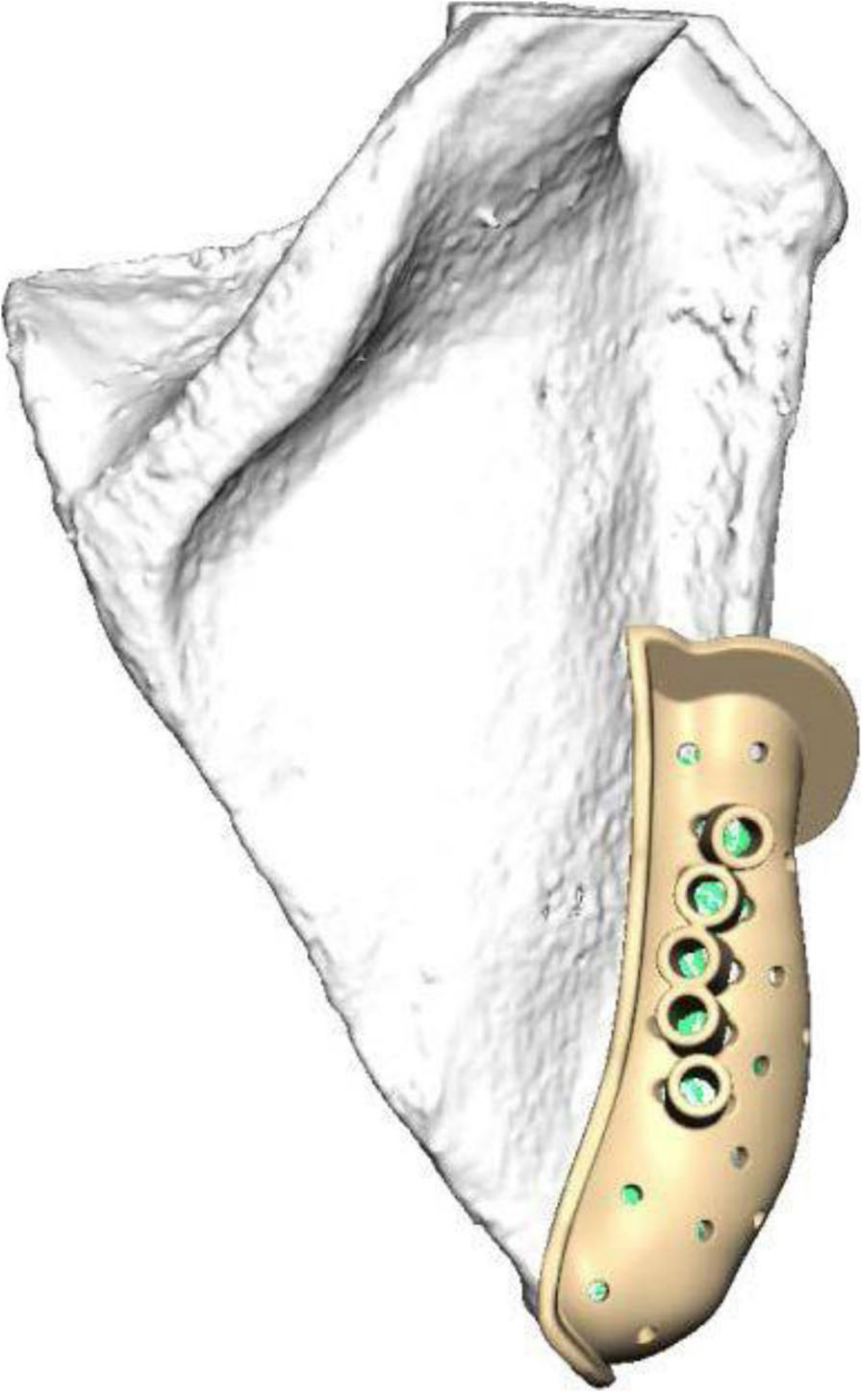
CAD constructed cutting and drilling guide for resection of scapula flap

To harvest the scapular flap, the patient is brought in a lateral position. The latissimus muscle is identified and accessed after outlining the desired cutaneous part of the transplant. This is followed by determining the arterial and venous branching pattern of the transplant and mobilization of the pedicle, which is based on the subscapular system. The lateral margin and the tip of the scapula are palpated and outlined. The bone segment is resected using the CAD/CAM fabricated cutting guide; the margin of the scapular tip becomes the new articular surface in the glenoid fossa (Fig. 3). The transplant vessels are cut after full mobilization of the osseomusculocutaneous flap.

To incorporate the transplant, the patient is placed in a supine position again. The patient specific osteosynthesis plate is fixed to the scapula flap, with drill holes placed using the guidance in the cutting guide (Fig. 2c). The osteosynthesis plate serves as a guidance for the placement of the scapula flap (Fig. 2d). The scapular tip is placed in the glenoid fossa. To suspend the neo-condyle into the glenoid fossa, 3 mersilene sutures (Ethicon, Cincinnati, Ohio, USA) are placed through holes drilled in the scapula tip and glenoid fossa.

Microvascular anastomoses are performed by hand suture using prolene 9–0 sutures (Ethicon, Cincinnati, Ohio, USA) under a surgical microscope (Leica MS3, Leica Microsystems, Germany). A Doppler probe (Cook Medical, Bloomington, Indiana, USA) is attached to the vein of the pedicle for postoperative flap monitoring. Soft tissue inset and wound closure are performed in the standard manner. Information on the size of the soft tissue flap and the recipient vessels used in each case is given in Table 1.

**Table 1:** Summary of clinical patient data and information on transplants and therapy

Patient No.	1	2	3
Age (years); sex	51; m	53; f	72; m
Diagnosis	Recurrent liposarcoma of left paroid gland	Basosquamous carcinoma of right cheek	Osteoradionecrosis (ORN)
Comorbidities	Essential hypertension	Chronic myelogenous leukemia; chron. Graft-versus-host-disease (liver, skin, mucosa)	
Length of scapular bone segment (mm)	82	67	79
Size of soft-tissue flap (cm)	7 × 12	12 × 14	10 × 12
Recipient vessels	External carotid artery (end-to-end) Superior thyroid vein (end-to-end)	External carotid artery (end-to-end) Internal jugular vein (end-to-side)	External carotid artery (end-to-end) Superior thyroid vein (end-to-end)
Adjuvant therapy	Intraoperative brachytherapy		
Follow-up (months)	17	16	2

In all patients, guiding elastics were incorporated for 7 to 10 days. Patients were fed via a nasogastric tube for 7 to 10 days and were recommended to have a soft diet the following 2 to 4 weeks. A postoperative CT scan was performed in all patients in postoperative week 1 or 2. After the patients’ discharge, postoperative follow-up examinations were performed in a 2 to 3 months interval.

At these examinations, patients were evaluated for postoperative complications with a focus set on malocclusion, limitations of mouth opening and plate loosening. The neo-TMJ was examined for bone resorption in the area of the glenoid fossa and the neo-condyle as well as signs of ankylosis on the basis of postoperative panoramic radiographs and CT/MRI scans.

**Fig. 4:**
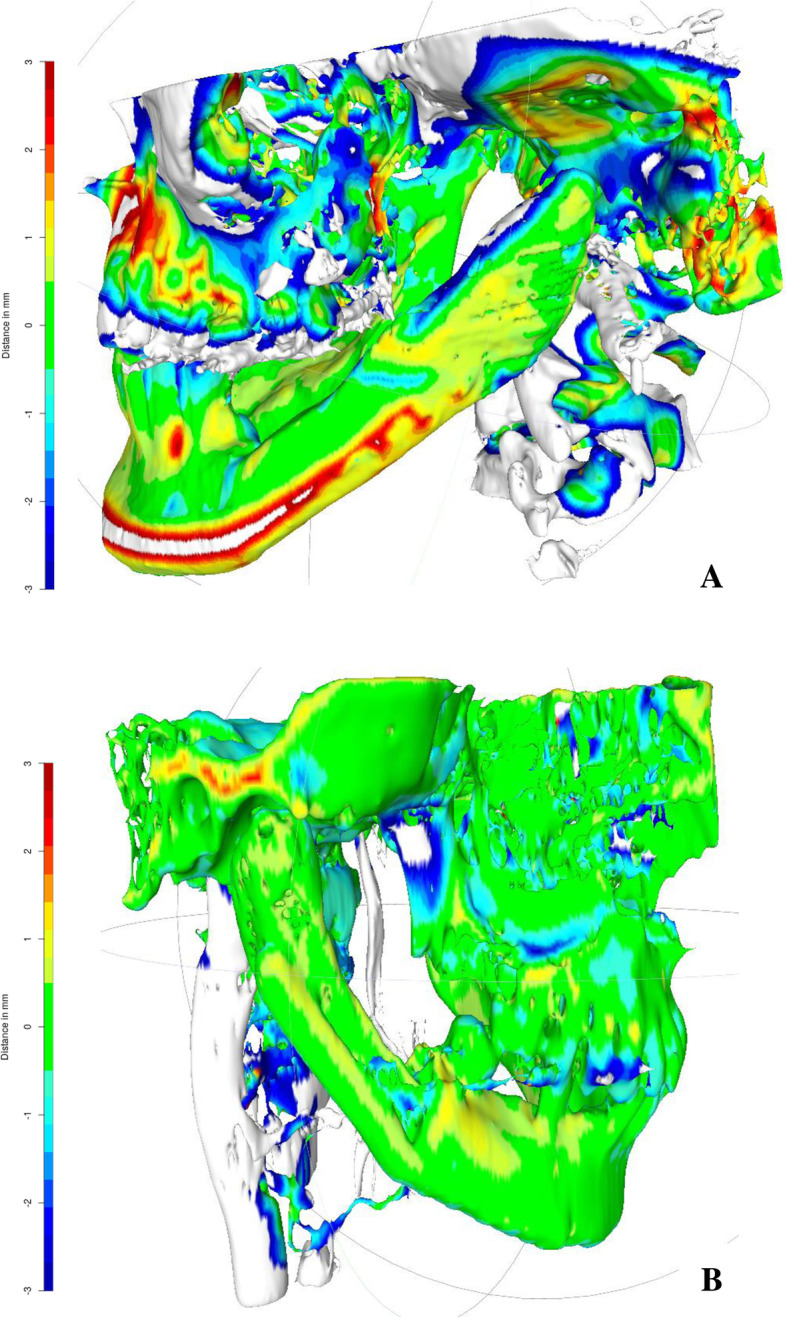
Heatmap created from CT scans performed in patient 1 at postoperative week 1 and postoperative month 12 (**a**) and patient 2 with CT scans performed at postoperative week 2 and postoperative month 7 (**b**). Local shape differences between the two CT scans are visualized by a color scale

Heatmaps were created from the postoperative CT scans to evaluate and visualize position stability of the neo-condyle. The open source software 3D Slicer was used for segmentation of pre- and postoperative CT scans, as well as the subsequent generation of 3D surface meshes. The resulting meshes were rigidly aligned employing a transformation based on anatomical landmarks (coronoid process, mandibular foramen and mental foramen). A closest point search was conducted to obtain per-vertex distances in order to create a heatmap representing local shape differences. The open source software MeshLab was used to visualize the resulting heatmaps (Fig. 4).

## Results

The patient’s clinical data is summarized in Table 1. Patients were followed up for 17 months (patient 1), 16 months (patient 2) and 2 months (patient 3).

The surgical procedure was successful in all cases. Although patient 3 had a postoperative follow-up of only 2 months, we decided to include this patient, as the complex surgical procedure was deemed successful and no complications occurred during the first 2 postoperative months.

Lengths of the reconstructed mandibular defects are given in Table 1. The reconstruction plate could be placed with good contact and postoperative panoramic radiograph and CT controls revealed the bone transplant to be positioned as preoperatively planned and with good long-term position stability of the neo-condyle.

**Fig. 5:**
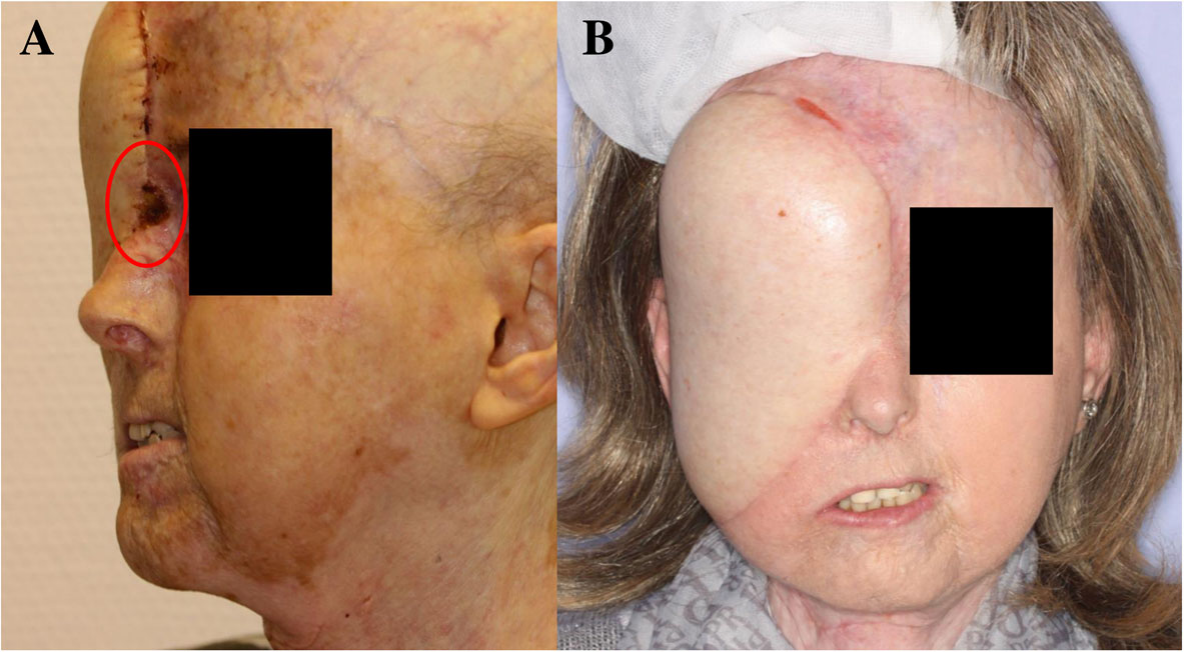
**a** Ulcerative skin alteration in patient 2 (marked with red circle) and conditions 9 weeks later (**b**)

**Fig. 6:**
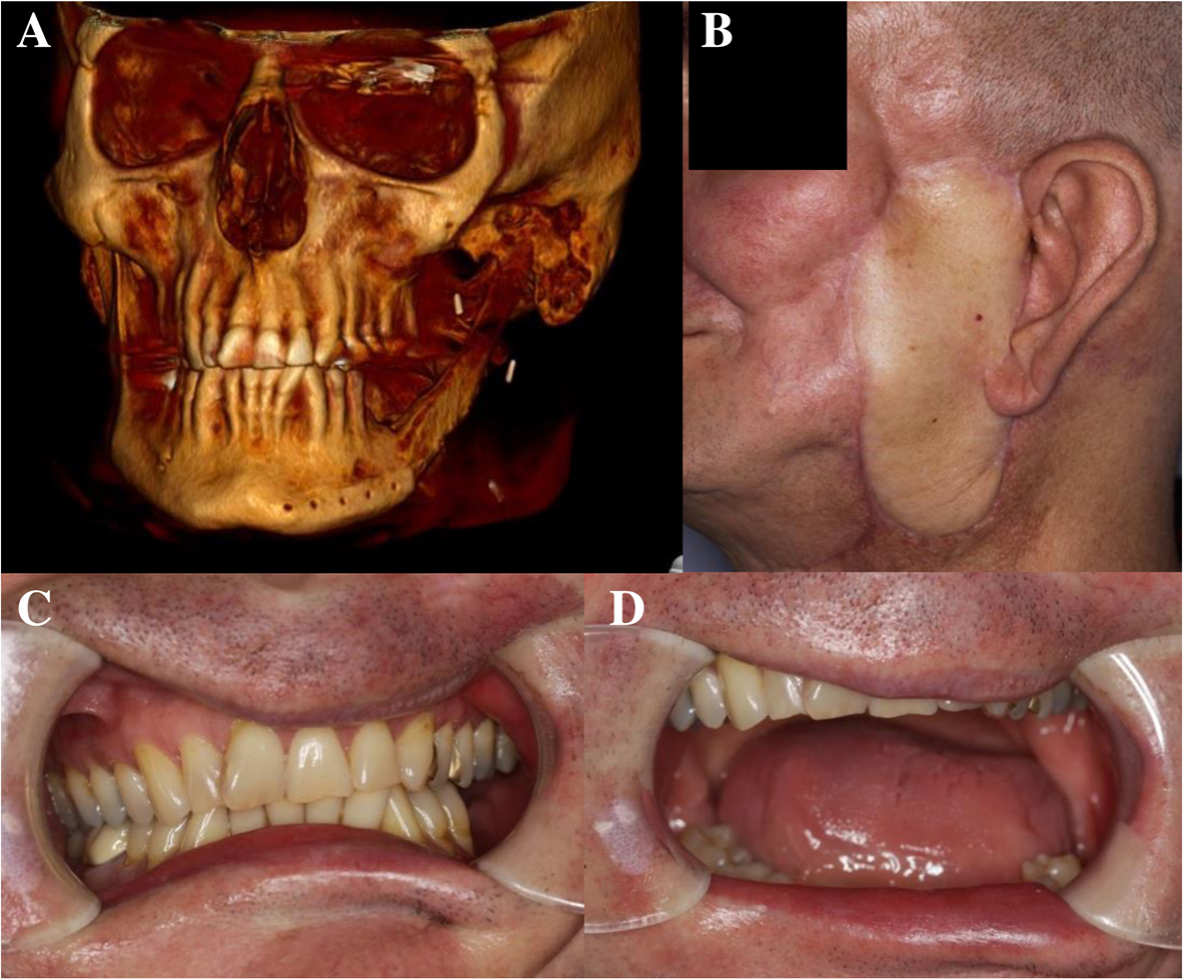
Postoperative CT-scan (**a**) and extra−/intraoral photographs (**b-d**) of patient 1

**Fig. 7:**
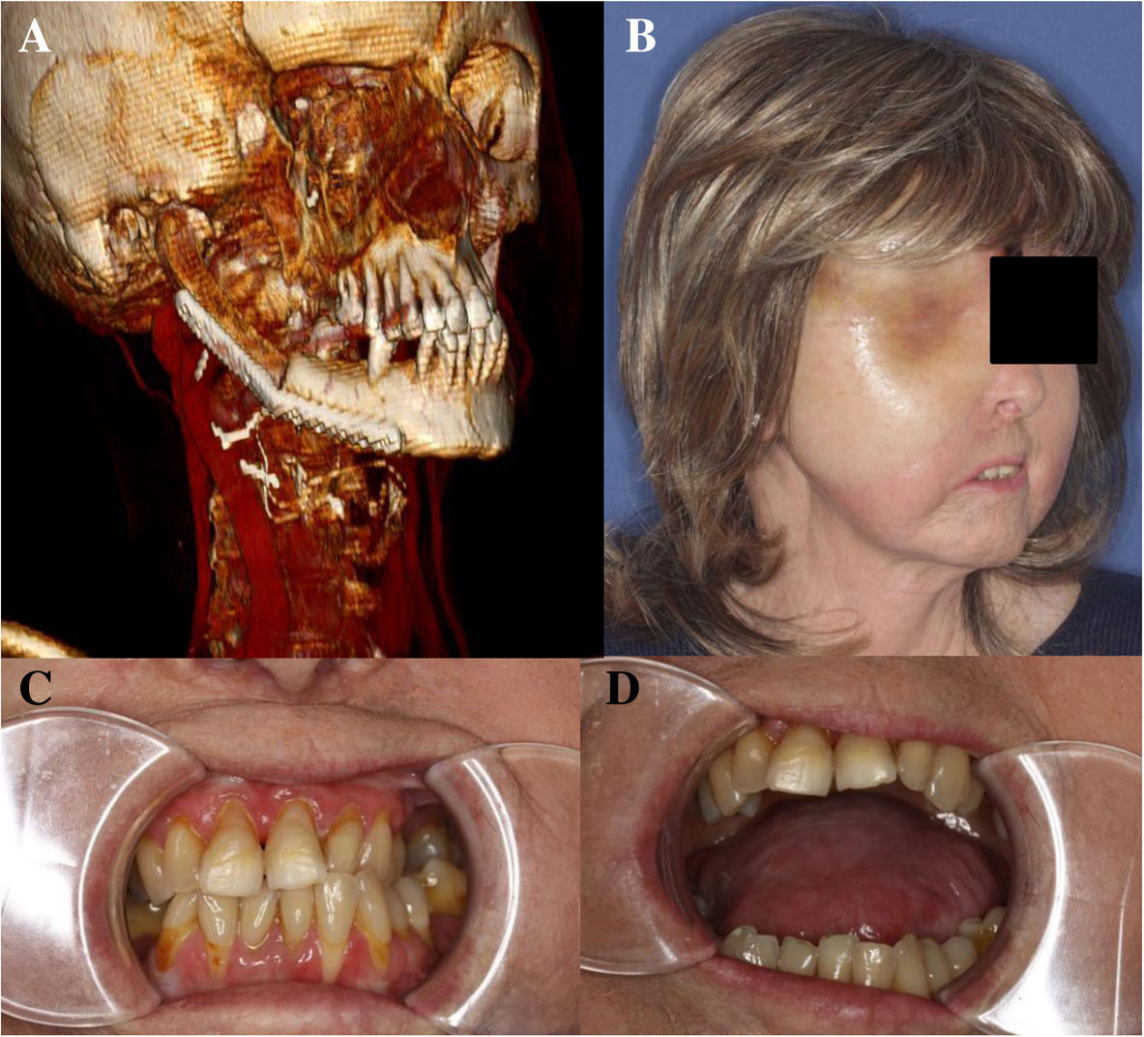
Postoperative CT-scan (**a**) and extra−/intraoral photographs (**b-d**) of patient 2

Intervention was required in patient 1 for venous congestion of the transplant on postoperative day 2. In a surgical intervention, the transplant vessels and anastomoses were accessed. Congestion could be successfully managed by surgically reducing the pressure of surrounding soft tissue onto the transplant vessels. Wound healing disturbance in the area between the latissimus transplant and the columella occurred in patient 2. The ulcerative skin alteration (Fig. 5a) was excised and submitted for histopathologic examination to rule out a malignant process. The excision wound healed by secondary intent. During follow-up, patient 1 underwent surgery for the suspicion of recurrent tumor. This suspicion was excluded via intraoperative histopathologic examination. During the intervention, the osteosynthesis plate was removed and sound union of the bone fragments could be observed (Fig. 6a). Patient 2 underwent further surgical intervention for anatomical re-contouring of the latissimus transplant 7 months after the primary surgical intervention (Figs. 5b and 7b).

All patients regained their preoperative occlusion pattern and subjectively satisfying mouth-opening ranges (Fig. 6c,d; Fig. 7c,d). Patient 1 and patient 3 reported postoperative TMJ pain and restricted mandibular mobility. However, these symptoms were only transient and lasted for less than 7 weeks in both cases. Regarding long-term subjective outcomes, all patients (including patient 3) stated to have no TMJ pain and reported to be not restricted in their diet. Adequate long-term joint mobility in terms of lateral, retrusive and protrusive movements with low-grade mandibular deviation during maximal mouth-opening was observed in patient 1 and patient 2.

**Fig. 8:**
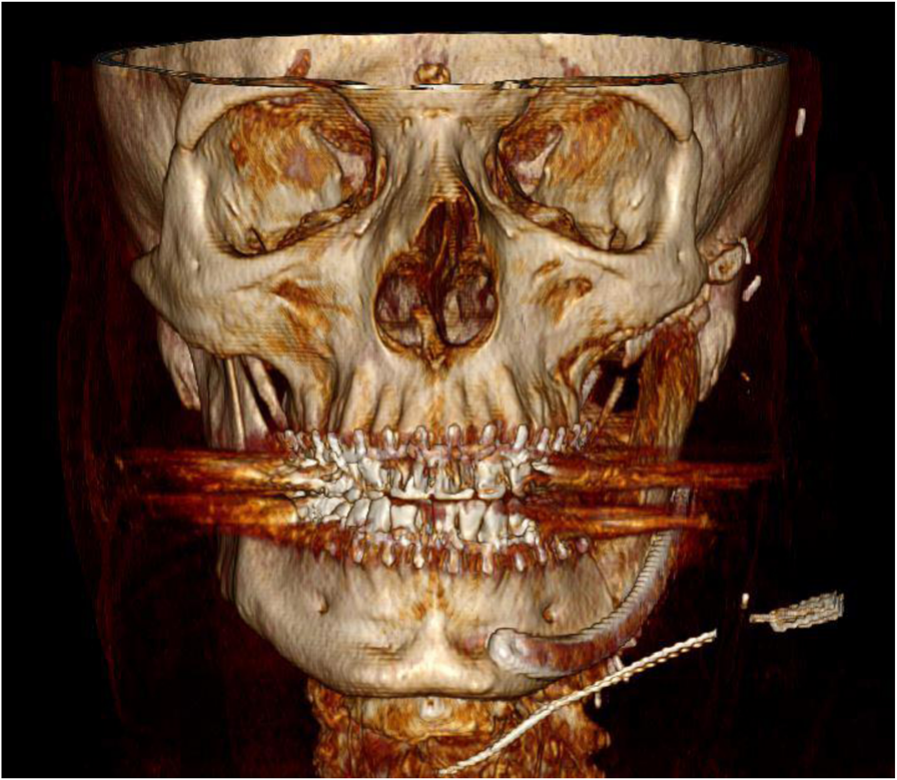
Postoperative CT scan of patient 3

A follow-up CT scan was performed in patient 1 at postoperative month 12 and in patient 2 at postoperative month 7. Postoperative radiograph controls confirmed a stable position of the neo-condyle in the glenoid fossa without evidence of bone resorption or ankylosis (Fig. 6a; Fig. 7a). There was no evidence of plate failure or loosening of screws in any of the patients. The CT scan of patient 3 performed at postoperative week 2 revealed the transplant to be positioned as preoperatively planned and without signs of loosening of the osteosynthesis plate (Fig. 8).

Timely wound healing of the donor site without any further donor site complications was observed in the patients. Regarding long-term subjective functional outcome, all patients stated not to be restricted in their shoulder mobility.

## Discussion

To our knowledge, this is the first article to report CAD/CAM planned reconstruction of the mandibular ramus and condyle with a vascularized scapula flap. Involvement of the mandibular condyle is not commonly seen in oncological processes originating from the oral cavity [11]. This finding may be attributed to the relative protective distance between most intraoral tumors and the mandibular condyle and correspondents to the findings in this article. In the patients presented, the involvement of the mandibular condyle resulted from cutaneous cancer, parotid tumor and ORN.

The vascularized scapula flap, first described for reconstructive measures in oral and maxillofacial surgery by Deraemaeker et al. in 1988 [17], is now well established in mandible and maxilla reconstruction. However, to date the application of this flap for reconstruction of the ascending ramus and condyle following disarticulation mandibular resection has only been reported in few cases [13, 18]. Virtually planned surgical procedures with the use of computer-aided design and manufacturing (CAD/CAM) are well established and reported to produce reliable results in mandible reconstruction [19, 20]. However, to our knowledge, to date this approach has not been reported for planning and performing reconstruction of the mandibular ramus and condyle using a scapula flap.

The major goal of reconstruction after resection of the mandible is restoration of form and function [21]. This is particularly challenging in cases of resection of the mandibular ramus and condyle.

The use of alloplastic condylar prostheses has been reported by a number of authors [1, 2, 6–8]. However, complications like hardware failure/degradation, foreign body reactions, implant exposure or erosion of the cranial base have been observed with this treatment approach [1]. Current literature reports varying complication incidence rates of approximately 10 to 25% for alloplastic condylar prostheses [6, 22, 23]. Moreover, radiation therapy, required by many oncology patients, alters the risk of postoperative plate exposure [11].

Reconstruction of the mandible and mandibular condylar process can be performed using a fibular single graft or fibular double-barrel vascularized graft [5, 24]. However, adequately shaping the fibula flap into the neo-mandible is a time consuming and challenging process, even more, if adequate three-dimensional shape of a neo-condyle has to be obtained as well [5, 25]. Some authors favor the integration of the native condyle into the mandibular reconstruction to obtain adequate TMJ-function [26]. However, this approach would not have been an option in any of the patients reported in this article.

Reconstructing mandibular defects, correct introduction and positioning of the flap in general and into the glenoid fossa in particular are of utmost importance to achieve satisfactory functional outcomes. As this can be very challenging by visual evaluation only, CAD/CAM planned procedures with the production of cutting guides and case specific osteosythesis plates provide surgeons with an option to achieve superior outcomes in reconstructing complex mandibular defects [27–29]. Applying various osteotomies to accurately shape the fibular bone into a neo-mandible might increase the risk of inaccuracy even in a CAD/CAM planned procedure [4].

The scapula flap possesses a number of advantages for reconstructive measures in general and for reconstruction of disarticulation resections in particular. The high volume of soft tissue provided by the latissimus dorsi flap was found to be highly beneficial for reconstructing the significant facial soft tissue defects present in all 3 patients. It was anticipated from the preoperative evaluation of the soft tissue involvement, that the resulting defects could not be reliably reconstructed using a combined fibula and radial forearm flap in these cases. Moreover, the shape and surface contour of the scapular tip seem like the ideal option to restore the shape of the mandibular condyle [13].

A technique for reconstructing large soft tissue defects in combination with reconstruction of the mandibular condyle may be required in a number of patients, as involvement of the condyle would result from extensive cutaneous tumors or aggressive parotid tumors rather than from (small) intraoral tumors. Moreover, this flap can be combined with a variety of different flaps from the subscapular arterial system like the serratus anterior, a split-latissimus or a parascapular flap. There is no consensus in the literature on the amount of bone that can be safely harvested using the angular scapular branch. However, by including the distal portion of the scapular lateral margin, Clark et al. [30] reported harvesting of bone lengths of up to 14 cm. In the cases reported in this article, length of the mandibular resection ranged from 67 to 82 mm and could reliably be reconstructed using a scapula bone flap. The major disadvantage of the described technique is the necessity of bringing the patient into a lateral position for harvesting of the flap. Thus a two-team approach is only practical in part for dissection of the vascular pedicle.

A major factor to be considered in the context of osteocutaneous free flap reconstruction is the potential of donor site morbidity. Complications reported for fibula free flaps include wound healing disturbances, sensory deficits in the area innervated by the superficial fibular nerve, (chronic) pain at the donor site and functional deficits (reduced ankle stability, gait deficits and reduced range of motion) [31]. It should be considered that sites requiring skin grafting, such as radial forearm flaps, can require prolonged periods to heal. This can potentially impact the patient’s quality of life and ability to undergo adjuvant treatment [32].

With the scapula free flap, reported donor site complications include wound healing disturbance and restriction of shoulder mobility/function; however, most investigators report only low long-term shoulder morbidity [33, 34].

Performing meta-analysis on donor site outcome is impeded by factors like varying outcome criteria of different reports and varying sizes of the harvested flaps. However, in a literature review comparing donor site morbidity of 4 commonly used osteocutaneous flaps in head and neck reconstruction, Kearns et al. found the scapula flap to have the lowest relative donor site morbidity [32].

The limitations of this report are the small number of patients included and intermediate/short follow-up periods. However, the results provide a basis for this technique to be investigated in a larger cohort. We recommend the technique described to be applied in cases with involvement of the mandibular condyle and significant amounts of soft tissue.

## Conclusion

This is the first article to report the feasibility of CAD/CAM assisted reconstruction of disarticulation defects using a scapula and latissimus dorsi free flap. Within a follow-up period of up to 17 months, we found a good functional and esthetic outcome in patients severely affected by cancer or ORN. No major complications occurred at the donor site and the site of reconstruction.

## Data Availability

All data supporting the findings of this article is included within the article.

## Abbreviations

CAD: Computer-aided design
CAM: Computer-aided manufacturing
CBCT: Cone beam computed tomography
CT: Computed tomography
ORN: Osteoradionecrosis
TMJ: Temporomandibular joint
